# Do insomnia and physical activity mediate the relationship between diabetes and depression in people with type 2 diabetes?

**DOI:** 10.1007/s40200-026-01875-x

**Published:** 2026-02-07

**Authors:** Sebastian Markvard Petersen, Daniel B. Ibsen, Anette Andersen

**Affiliations:** 1 Steno Diabetes Center Aarhus, Aarhus University Hospital, Palle Juul-Jensens Boulevard 11, Aarhus N, 8200 Denmark; 2 Department of Public Health, Aarhus University, Bartholins Allé 2, Aarhus C, 8000 Denmark

**Keywords:** Depression, Diabetes, Type 2, Insomnia, Physical activity, Mediation

## Abstract

**Purpose:**

To examine how self-reported depressive symptoms are affected by type 2 diabetes, and how this relationship may be mediated by insomnia and physical activity.

**Methods:**

We used cross-sectional data from the Health In Central Denmark (HICD) cohort including persons with and without type 2 diabetes aged 18 to 75 years. Register-identified diabetes status was used as the exposure and The World Health Organization Five Well-Being Index (WHO-5) was used as the outcome measure for risk of depression. Insomnia severity index level and physical activity level were used as potential mediators. We performed logistic regression analyses and cross-sectional mediation analyses.

**Results:**

In total, 45,988 participants were included. Of these, 19,907 had type 2 diabetes of which 1,396 were at risk of depression. Adjusted odds ratio (OR) for risk of depression in people with type 2 diabetes was 1.55 (95% confidence interval (CI): 1.19, 1.59). The strongest risk factor for depression was insomnia at an adjusted OR of 7.43 (CI: 6.63, 8.32). The relationship between diabetes and risk of depression may be mediated by both insomnia and physical activity, the proportion mediated being 42.1% for insomnia and 20.5% for physical activity, in our analyses.

**Conclusion:**

Living with type 2 diabetes was associated with higher odds of depression, and insomnia and physical activity may mediate this relationship. Insomnia and physical activity may be important factors for healthcare professionals to consider in their treatment of people with type 2 diabetes.

## Introduction

Mental health conditions, such as depression, are prevalent in people with diabetes [1, 2]. Mental health substantially influences diabetes care, with self-managing diabetes being extremely complex for patients, who also have severe mental illness [3]. A systematic review found that diabetes is associated with a marked increase in suicidal behavior and suicidal ideation, especially in patients with depressive symptoms, with unsatisfactory glycemic control being among the risk factors [4]. In a study involving 216 adults with type 2 diabetes, 41% reported elevated symptoms of depression [1]. A meta-analysis of 42 studies found that the odds of depression, assessed by Diagnostic Interview Schedule or Structured Clinical Interview for Diagnostic and Statistical Manual of Mental Disorders, 3rd Edition, Revised (DSM-III-R), among people with diabetes is twice that of people without diabetes and did not differ by type of diabetes [2]. Type 2 diabetes is a progressive condition that tends to get worse over time [5]. Therefore, it is important to identify modifiable risk factors for depression in people with diabetes to promote better self-management and longer lifespan.

Two key modifiable risk factors related to type 2 diabetes and depression are disturbed sleep, especially insomnia, and physical activity. Disturbed sleep is a core feature of depression, with up to 90% of depressed individuals having sleep problems, and it is a key risk factor for the onset and recurrence of depression [6]. Previous research on modifiable risk factors included a cohort study of 232,489 people with the aim of examining the influence of comorbid sleep disorder on the association between type 2 diabetes and risk of depression. The study suggests that type 2 diabetes and sleep disorders are independently associated with subsequent risk of depression and individuals with both conditions experience the greatest relative risk [6]. Physical activity is also associated with both diabetes and depression [6]. A meta-analysis of 81 studies found a strong inverse relationship between physical activity and risk of type 2 diabetes [7]. Another meta-analysis of 49 studies found that, compared to people with low levels of physical activity, those with high levels had a lower odds ratio (OR = 0.83) of developing depression [8]. Physical activity therefore could be an important confounder in the relationship between type 2 diabetes and risk of depression.

The present study focused on modifiable risk factors, such as insomnia and physical activity, using a dataset with self-reported responses and register based data from 51,853 persons with and without diabetes. While correlations have previously been observed for these variables, to our knowledge, it has yet to be determined whether the association between diabetes and depression is mediated by insomnia and physical activity. Therefore, the purpose of the present study was to examine how self-reported depressive symptoms, measured by The World Health Organization Five Well-Being Index (WHO-5), are affected by diabetes, and how this relationship may be mediated by insomnia and physical activity, in people with type 2 diabetes.

## Materials and methods

### Data sources and study population

The present study used the Health in Central Denmark (HICD) cohort [9]. HICD encompasses health information about 51,853 persons residing in the Central Denmark region, between the ages of 18 and 75 years old, with type 2 diabetes (*n* = 19,907) and without diabetes (*n* = 26,081). All available cases were included. The study population was matched by gender, age, and municipality by random sample, i.e. the matched group without diabetes was not matched directly with an individual with diabetes. The matched group without diabetes was drawn from the entire Danish population, rather than only the population of the Central Denmark Region as in the original group, limiting selection bias. The data was obtained from a 90-item questionnaire electronically distributed in 2020. Routinely collected Danish national health registry data on hospital diagnoses, medication use and socioeconomic status from decades prior as well as laboratory data with repeated measures of biochemical markers, such as glycated hemoglobin, up to a decade prior was linked to the questionnaire data. The questionnaire included data about patient experiences on how their medical conditions cause daily life challenges that are not objectively measured in clinical settings [9]. 

For the purpose of this study, people with types of diabetes other than type 2 (*n* = 3,331) and participants who did not respond to the outcome measure (WHO-5) (*n* = 2,534) have been omitted, leaving 45,988 persons in this study. Complete case analysis was used.

### Measures, determinants and outcome

As an outcome measure, we used the WHO-5 wellbeing index, which has proven to be an acceptable screening instrument for risk of depression in people with diabetes [10]. WHO-5 is a battery of 5 questions, including whether one has felt cheerful and in good spirits, and if one’s daily life has been filled with things of interest, in the past 2 weeks. Response categories range from “all the time” to “at no time”. Each of the 5 questions has 6 possible answers, each with an assigned point value ranging from 0 to 5. A sumscore is calculated by adding the point values, range from 0 to 25, multiplied by 4, making the range 0 to 100. The cut-off point for poor emotional well-being is 50 or less, 28 or less is a cut-off point for risk of depression, the latter of which was used in the present study [11]. 

The determinant was diabetes type based on registry data which defined participants with type 2 diabetes and participants without diabetes. This variable was based on an algorithm based on diagnosis and medication purchases. People were included in the type 2 diabetes category, if their last type-specific diabetes diagnosis was type 2, and if they have purchased non-insulin glucose-lowering drugs. People were excluded from the category: If they were women with any polycystic ovary syndrome related drugs or diagnoses. If there was only one inclusion event. Or if there were no non-insulin glucose-lowering drugs purchases or type 2 diabetes diagnoses within 10 years prior to index date [12]. 

We included mediators from the HICD survey: Insomnia and physical activity. Insomnia severity index level is a sumscore based on 7 items: Difficulty falling asleep, difficulty staying asleep, problem waking up too early, satisfied/dissatisfied with sleep pattern, sleep problem interferes with daily function, noticeable to others, and worried/distressed about current sleeping problem. Each item is rated by a 5-point Likert scale, answers ranging from “none” to “very severe”, from “not at all” to “very much”, and from “very satisfied” to “very dissatisfied”, points ranging from 0 to 4, allowing for a maximum score of 28. The sumscore is divided into categories: 0 to 7 is absence of insomnia, 8 to 14 is subthreshold insomnia, 15 to 21 is moderate insomnia, and 22 to 28 is severe insomnia [13]. In this study, this variable has been dichotomized into “no/subthreshold insomnia” ( < = 14) and “moderate/severe insomnia” ( > = 15). Physical activity in leisure time included 4 possible answers ranging from “regular hard physical training” to “sedentary”, which in this study have been dichotomized into “regular physical activity” and “less than regular physical activity”. These and other variables have been dichotomized to simplify the statistical analyses for easy interpretation and presentation of results.

Potential confounders were selected based on a review of the literature. Confounding variables from the HICD survey were: Smoking, which comprised 5 possible response categories ranging from “daily” to “never”, dichotomized into smoking currently or not. Alcohol consumption also comprised 5 possible answers ranging from “never” to “4 or more times a week”, dichotomized into “2–4 times a month or less” and “2–3 times a week or more”. Loneliness comprised 4 possible answers ranging from “no” to “often”, and was dichotomized into “no or rarely” and “once in a while or often”. Confounding variables included from registry data were: Sex, which in this study was binary. Age at the time of the survey which was continuous. Employment status, dichotomized into currently employed or not. Marital status, dichotomized into currently partnered or not. Migration status was dichotomized into Danish and non-Danish origin, with migrants and descendants thereof being categorized as having non-Danish origin, based on their birth country and that of their parents. Mean glycated hemoglobin (HbA1c) measures from 2020, which was dichotomized into below 48 mmol/mol and 48 mmol/mol and above. All of these potential confounding variables have been associated with depression [4, 14–26], insomnia [27–35] and physical activity [36–44], and hence all of these confounding variables were included in each analysis. We also controlled for collinearity between the two mediating variables, as previous research has found that physical activity improves sleep which, in turn, reduces depression [45]. 

### Statistical analyses

First, we did descriptive statistics, using the “basetable” command in Stata, creating an overview table for all of the variables by diabetes status.

Then, we performed logistic regression analyses in Stata. First investigating the association between diabetes status and depression, without any confounding variables, to get a crude OR, then with all of the confounding variables to get an adjusted OR and determine whether there was an overall association that merited further investigation by mediation analysis.

Cross-sectional mediation analyses were also performed in Stata using the “mediate” command which estimates: The natural indirect effect (NIE) of the mediator variable, i.e. how much of the relationship between the determinant and outcome variables can be explained with the mediating variable. The natural direct effect (NDE) of the determinant variable, i.e. how much of the relationship between the determinant and outcome variables can be explained by the determinant variable independently of the mediating variable. And the total effect (TE) of both mediator and determinant variables on the outcome variable, in this case using a logistic model. The proportion mediated (PM) is NIE divided by TE, multiplied by 100 for percentage. ORs were derived from log odds by taking the exponential (exp) using the estat command.

Many of the included variables had no missing data at all, as they were based on registry data. However, the variable with the highest missing percentage was HbA1c at 40.3%. This was mostly due to over triply as many people without diabetes not having been tested for HbA1c, compared to people with type 2 diabetes (Table 1). To remedy this, these missings were changed into their own response category, “Not tested”, because the alternative of deleting so many missings, which were known to not be missing completely at random, would likely introduce bias. We argue that “Not tested” is a valid and natural category in this case, as the missings are due to many people not having been tested for HbA1c in this study. The variable with the second highest missing percentage was insomnia severity index level at only 1.54%. The rest of the missing data were excluded.

**Fig. 1:**
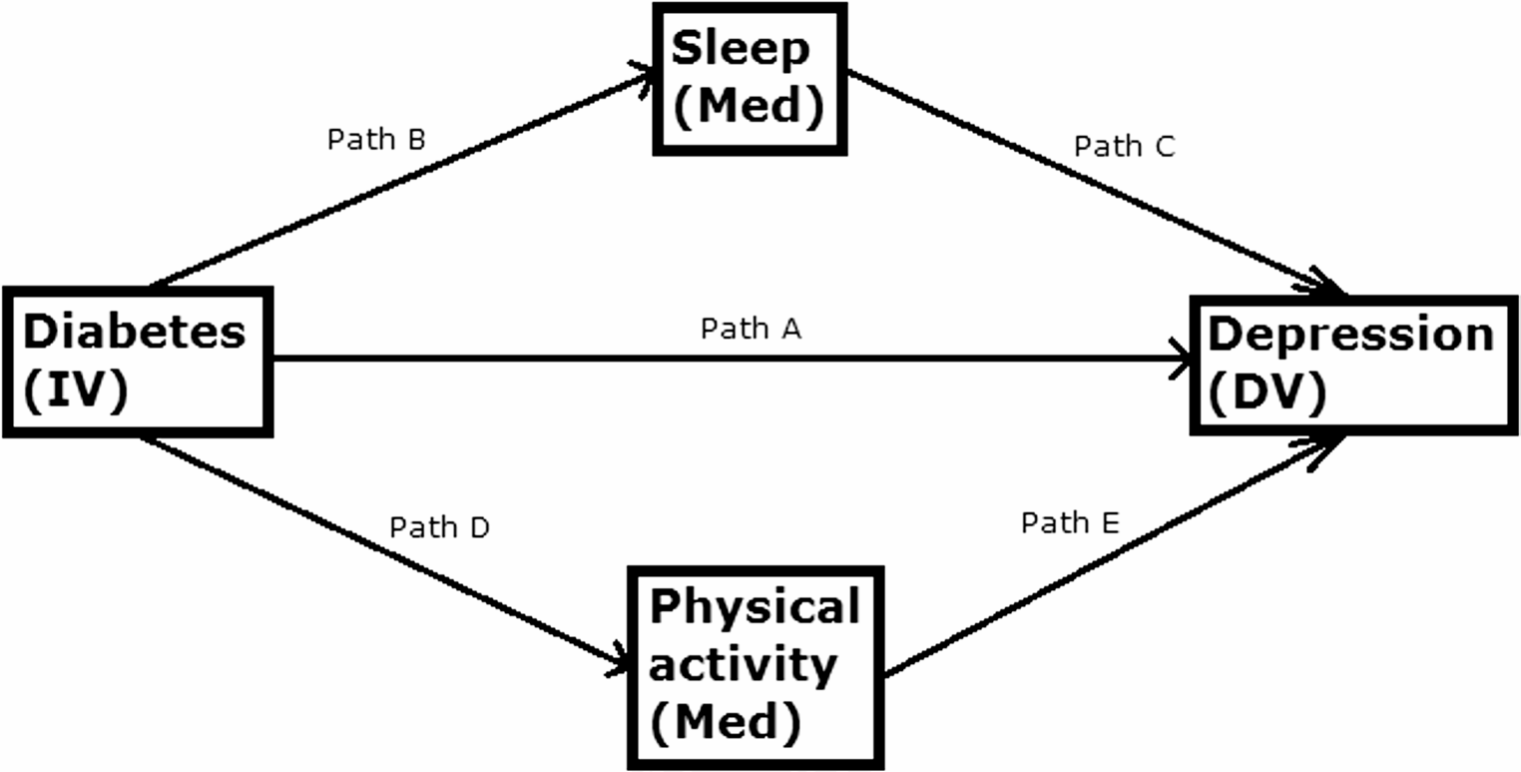
Plan of analysis (IV is the independent variable, DV is the dependent variable, Med are the mediating variables)

First, a logistic regression analysis was performed, testing how diabetes affects risk of depression (Path A), and so on for paths B, C, D and E (Fig. 1). Then mediation analyses were performed, first testing how the relationship between diabetes and risk of depression is mediated by insomnia (Path B-C), then how the relationship is mediated by physical activity (Path D-E) (Fig. 1).

### Ethics

According to Danish law, ethical approval of questionnaire and register based studies is not required. The cohort is registered in the internal register of research projects in the Central Denmark Region. All analyses were made on the remote server of Statistics Denmark, always ensuring confidentiality.

## Results

**Table 1:** Summary table (*n* = 45,988) for differences between persons with type 2 diabetes and persons without diabetes

Diabetes	Persons without diabetes	Persons with type 2 diabetes	Total	Missings / *N* (Pct)
Number of participants, n (%)	26,081 (100.0)	19,907 (100.0)	45,988 (100.0)	0 / 45,988 (0.00)
Sex, n (%)				
Male	14,705 (56.4)	11,755 (59.0)	26,460 (57.5)	
Female	11,376 (43.6)	8152 (41.0)	19,528 (42.5)	0 / 45,988 (0.00)
Age, mean (sd)	62.2 (11.6)	63.6 (9.8)	62.8 (10.8)	0 / 45,988 (0.00)
Smoking status, n (%)				
Doesn’t smoke currently	22,034 (84.5)	16,167 (81.2)	38,201 (83.1)	
Smokes currently	3785 (14.5)	3501 (17.6)	7286 (15.8)	501 / 45,988 (1.09)
Alcohol consumption, n (%)				
2–4 times a month or less	12,875 (49.4)	13,819 (69.4)	26,694 (58.0)	
2–3 times a week or more	13,003 (49.9)	5904 (29.7)	18,907 (41.1)	387 / 45,988 (0.84)
Leisure time activity level, n (%)				
Regular physical activity	6166 (23.6)	2492 (12.5)	8658 (18.8)	
Less than regular physical activity	19,852 (76.1)	17,327 (87.0)	37,179 (80.8)	151 / 45,988 (0.33)
Feeling lonely, n (%)				
No or rarely	19,937 (76.4)	13,776 (69.2)	33,713 (73.3)	
Once in a while or often	5778 (22.2)	5856 (29.4)	11,634 (25.3)	641 / 45,988 (1.39)
WHO-5 score, n (%)				
Not at risk of depression ( > = 28)	25,122 (96.3)	18,511 (93.0)	43,633 (94.9)	
At risk of depression (< 28)	959 (3.7)	1396 (7.0)	2355 (5.1)	0 / 45,988 (0.00)
Insomnia severity index level, n (%)				
No/subthreshold insomnia	23,088 (88.5)	16,301 (81.9)	39,389 (85.7)	
Moderate/severe insomnia	2601 (10.0)	3291 (16.5)	5892 (12.8)	707 / 45,988 (1.54)
Marital status, n (%)				
Partnered	17,981 (68.9)	12,691 (63.8)	30,672 (66.7)	
Not partnered	8100 (31.1)	7216 (36.2)	15,316 (33.3)	0 / 45,988 (0.00)
Migration status, n (%)				
Danish	24,868 (95.3)	18,288 (91.9)	43,156 (93.8)	
Not Danish	1213 (4.7)	1619 (8.1)	2832 (6.2)	0 / 45,988 (0.00)
Employment status, n (%)				
Employed	14,025 (53.8)	8105 (40.7)	22,130 (48.1)	
Unemployed	12,056 (46.2)	11,802 (59.3)	23,858 (51.9)	0 / 45,988 (0.00)
HbA1c, n (% )				
<48 mmol/mol	10,729 (41.1)	6030 (30.3)	16,759 (36.4)	
>=48 mol/mol	46 (0.2)	10,652 (53.5)	10,698 (23.3)	
Missing/not tested	15,306 (58.7)	3225 (16.2)	18,531 (40.3)	0 / 45,988 (0.00)

In total, we included 45,988 persons where 43.3% had type 2 diabetes (Table 1). People with type 2 diabetes are more likely to be at risk of depression, measured by WHO-5 score below 28, as 7.0% of them are at risk of depression compared to 3.7% of people without diabetes (Table 1).

**Table 2:** Logistic regression analysis (*n* = 44,760) with diabetes as the determinant and risk of depression, measured by WHO-5 score, as the outcome. Crude and adjusted odds ratios, 95% confidence interval

	Crude odds ratio (95% confidence interval (CI))	*p*-value	Adjusted odds ratio (95% confidence interval (CI))	p-value
Type 2 diabetes	1.98 (1.82, 2.15)	0.000	1.55 (1.42, 1.70)	0.000

Adjusted for smoking, alcohol consumption, loneliness, sex, age, employment status, marital status, migrant status and HbA1c.

The crude OR for diabetes was 1.98 (95% confidence interval (CI): 1.82–2.15) (Table 2). Adjusting for background factors, health behaviors, loneliness and HbA1c decreased the OR for diabetes to 1.55 (CI:1.42–1.70) (Table 2).

**Table 3:** Logistic regression analysis (*n* = 44,514) for paths B, C, D and E. Crude and adjusted odds ratios, 95% confidence interval (CI)

Path	Exposure	Outcome	Crude odds ratio (95% CI)	*p*-value	Adjusted odds ratio (95% CI)	*p*-value
B	Type 2 diabetes	Insomnia	1.79 (1.70, 1.89)	0.000	1.54 (1.45, 1.64)	0.000
C	Insomnia	Depression	10.85 (9.93, 11.85)	0.000	7.67 (6.98, 8.42)	0.000
D	Type 2 diabetes	Lack of physical activity	2.16 (2.05, 2.27)	0.000	1.95 (1.85, 2.06)	0.000
E	Lack of physical activity	Depression	3.08 (2.63, 3.60)	0.000	2.62 (2.22, 3.10)	0.000

Adjusted for smoking, alcohol consumption, loneliness, sex, age, employment status, marital status, migrant status and HbA1c.

**Fig. 2:**
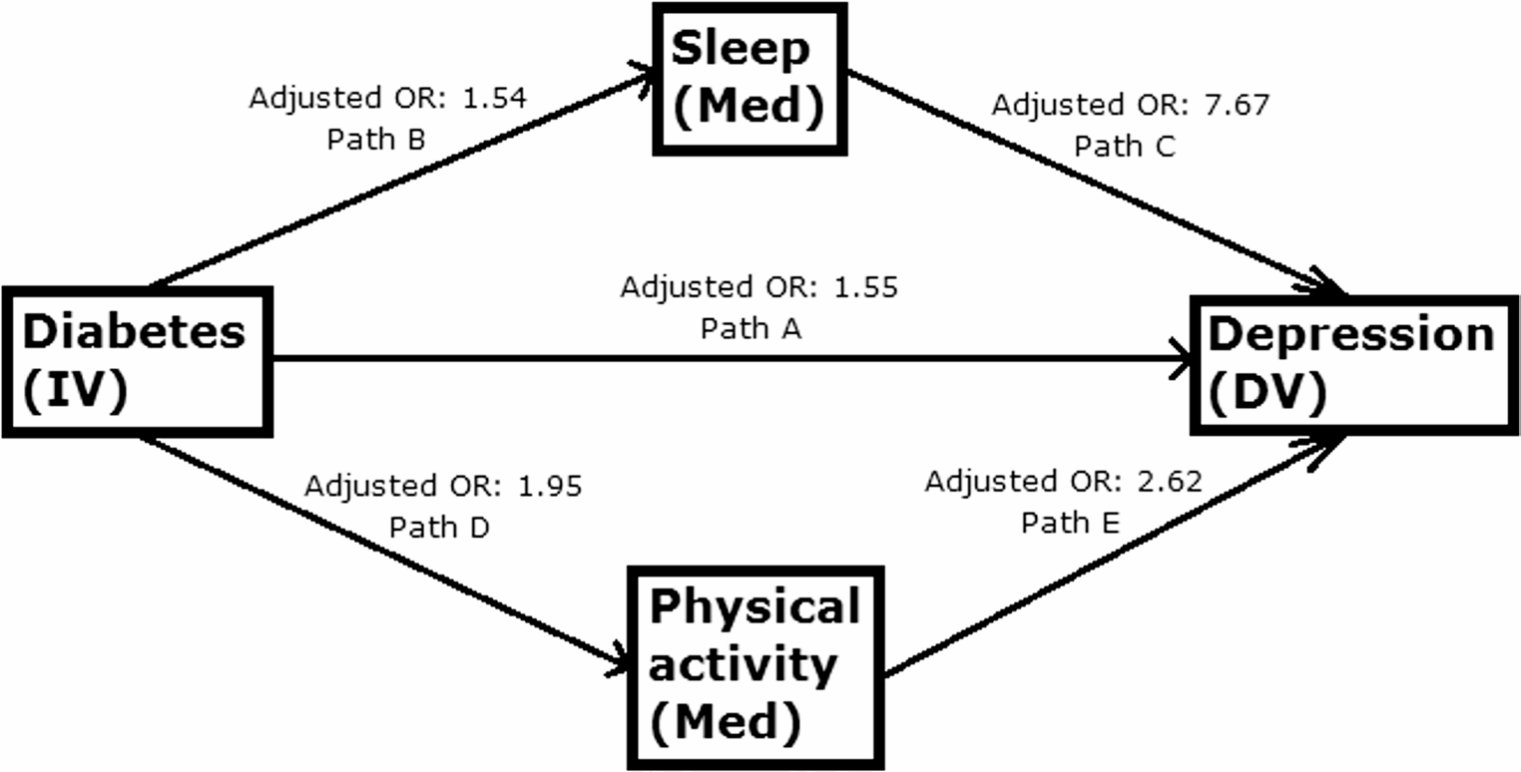
Adjusted ORs for each path based on logistic regression analysis

In analyses adjusted for background factors, health behaviors, loneliness and HbA1c, an association between diabetes and insomnia, and insomnia and depression, was observed (Table 3; Fig. 2). Also, diabetes was associated with physical activity and physical activity was associated with depression (Table 3; Fig. 2).

**Table 4:** Cross-sectional mediation analysis (*n* = 44,392) with diabetes as the determinant, risk of depression as the outcome and insomnia, measured by insomnia severity index level, as the mediator

	Coefficient/log odds (95% CI)	Crude OR (95% CI)	*p*-value	Coefficient/log odds (95% CI, controlling for confounders)	Adjusted OR (95% CI)	*p*-value
NIE	0.014 (0.012, 0.016)	1.27 (1.24, 1.31)	0.000	0.0072 (0.0059, 0.0085)	1.15 (1.12, 1.18)	0.000
NDE	0.019 (0.016, 0.023)	1.56 (1.44, 1.70)	0.000	0.0099 (0.0061, 0.0137)	1.25 (1.15, 1.36)	0.000
TE	0.033 (0.029, 0.037)	1.99 (1.83, 2.17)	0.000	0.0171 (0.0130, 0.0213)	1.44 (1.32, 1.57)	0.000
PM	42.4%			42.1%		

Adjusted for smoking, alcohol consumption, loneliness, sex, age, employment status, marital status, migrant status, HbA1c and physical activity.

Adjusting for background factors, health behaviors, loneliness, HbA1c, and physical activity, the natural indirect effect (NIE) of insomnia on the relationship between diabetes and risk of depression was 0.0072, and the total effect (TE) was 0.0171 (Table 4). Dividing the NIE with the TE and multiplying by 100 yields the proportion mediated (PM) percentage, which is 42.1%, meaning that insomnia may mediate the association between diabetes and risk of depression in this model (Table 4). ORs were derived from log odds by taking the exponential (exp).

**Table 5:** Cross-sectional mediation analysis (*n* = 44,392) with diabetes as the determinant, risk of depression as the outcome and physical activity, measured by leisure time activity level, as the mediator

	Coefficient/log odds (95% CI)	Crude OR (95% CI)	*p*-value	Coefficient/log odds (95% CI, controlling for confounders)	Adjusted OR (95% CI)	*p*-value
NIE	0.0054 (0.0045, 0.0063)	1.09 (1.08, 1.11)	0.000	0.0025 (0.0019, 0.0030)	1.05 (1.04, 1.06)	0.000
NDE	0.0274 (0.0233, 0.0314)	1.80 (1.65, 1.96)	0.000	0.0097 (0.0057, 0.0137)	1.23 (1.13, 1.35)	0.000
TE	0.0328 (0.0286, 0.0370)	1.96 (1.80, 2.14)	0.000	0.0122 (0.0081, 0.0162)	1.29 (1.19, 1.41)	0.000
PM	16.5%			20.5%		

Adjusted for smoking, alcohol consumption, loneliness, sex, age, employment status, marital status, migrant status, HbA1c and insomnia.

Adjusting for background factors, health behaviors, loneliness, HbA1c, and insomnia, the natural indirect effect (NIE) of a lack of physical activity on the association between diabetes and risk of depression was 0.0025, and the total effect (TE) was 0.0122 (Table 5). Dividing the NIE with the TE and multiplying by 100 yields the proportion mediated (PM) percentage, which is 20.5%, meaning that lack of physical may mediate the association between diabetes and risk of depression in this model (Table 5). ORs were derived from log odds by taking the exponential (exp).

## Discussion

### Main results

This study had 2 main results. First, we found that people with type 2 diabetes have a higher adjusted odds ratio (OR = 1.55) of being at risk of depression compared to people without diabetes, even after controlling for confounding variables. Though the largest risk factor for depression was insomnia with an adjusted OR almost 5 times higher (OR = 7.67). Second, we found that insomnia and lack of physical activity mediate the relationship between diabetes and risk of depression, however, insomnia (PM = 42.1%) did so to a higher degree than lack of physical activity (PM = 20.5%) in their respective models.

### Comparison to other studies

We found that type 2 diabetes increases the risk of depression almost twofold which is in line with previous research, such as the meta-analysis of 42 studies [2]. 

Unlike previous research, which found that sleep neither explained nor amplified the relationship between diabetes and depression after adjusting for several confounders, some of which were similar to our own, and some of which were various somatic and psychiatric conditions [6], we found that insomnia may mediate the effect of diabetes on risk of depression. Sleep education has been demonstrated to improve sleep and health outcomes in people with type 2 diabetes [46]. 

Previous research found that physical activity was associated with both diabetes and depression [6–8]. We found that physical activity mediated the relationship between diabetes and risk of depression, albeit to a smaller degree than insomnia. Previous research has confirmed that sleep and physical activity are treatable risk factors [46–48]. Physical activity has been demonstrated to be an effective medicine in the treatment of several diseases, including type 2 diabetes [47], improving both mental and physical health in people with depression [48]. 

### Strengths and limitations

The HICD survey is an ongoing research resource encompassing more than 50 thousand people, representing a heterogeneous population. It combines questionnaire data with routinely collected, nationwide registry data and laboratory data, focused on patient-related health information. With so many respondents, it is possible to look into details in specific subgroups of the population, as has been done in the present study by controlling for confounding variables, such as the almost 3 thousand migrants and descendants thereof [9]. The HICD survey provides many variables that could be used as confounders for controlled analyses, and it has enabled us to use a large combination of control variables, including migrant status, which, to our knowledge, is not commonly used in regards to depression, despite the established connection between the two [26]. However, one important health behavior factor that could be a confounder is diet, which was not available in this iteration of the HICD survey. A Mediterranean diet has been demonstrated to reduce depression [49]. Previous research has noted underlying mechanisms for the relationship between diabetes and depression, including insulin dysfunction, increased inflammation, and neuronal alterations, which were not included in this study [6]. Psychotropic medication use, diagnosed psychiatric comorbidities, and sleep apnea might also be potential confounders, which were not included here. Although we did control for substance abuse, e.g. alcohol and smoking, we were not able to fully control for “male-typical” symptoms of depression, such as overworking, substance misuse, and aggression, which are known to make the gender disparity disappear [20]. However, a strength of this study is that we controlled for confounders both between exposure-mediator and mediator-outcome.

Another strength of the present study is the mediation analyses, examining the indirect effects of insomnia and physical activity, rather than the direct effects as in several other studies [8, 50]. Otherwise one risks underestimating the total, combined effect of the direct and indirect effects of insomnia and physical activity respectively. To our knowledge, only one other study [6] has done this. Because the variable for diabetes type was register-based, and the variable for depression was based on a recent survey, it is probable that, temporally, diabetes came before depression. With that being said, we acknowledge that we cannot by design verify this, as we cannot rule out the possibility of reverse causation, because the only temporal variable available in HICD was time of diagnosis. However, a meta-analysis of longitudinal studies found that diabetes is associated with a significantly increased risk of depressive symptoms [51]. And a cohort study found that sleep disorders are associated with subsequent risk of depression [6]. So the temporality of these associations have been demonstrated elsewhere. Another strength of the present study is that we controlled for collinearity between the mediating variables, which, to our knowledge, is uncommon, yet justified in this case, as collinearity has previously been observed between sleep and physical activity [45]. In our data, there was a statistically significant correlation between insomnia and physical activity, however, it wasn’t particularly high (OR = 1.83, *p* = 0.000). Controlling for this collinearity is sufficient and does not bias the results of the mediation analysis, as an OR below 5 doesn’t indicate a problematic collinearity [52]. 

This study relied on register-based data to identify diabetes status. However, 44.7% of diabetes cases are undiagnosed, especially among those with low income [53]. So we cannot rule out the possibility that some of the people identified herein as people without diabetes might actually be undiagnosed people with diabetes, thereby introducing bias into the study, underestimating the differences between people without diabetes and people with type 2 diabetes.

The present study focused on treatable risk factors for depression, specifically in people with type 2 diabetes, however, some risk factors are not so easily treatable or changed, as is the case with personality. Certain personality traits are associated with depression. High neuroticism, low extraversion, and low conscientiousness are associated with the presence of a depression diagnosis and severity of depression, and high openness is associated with earlier onset of depression [54]. Research has suggested that depression does not change people’s personalities [55]. This implies that certain personality traits predispose people towards developing depression. Further research could examine how personality traits predispose people with type 2 diabetes towards certain health behaviors that might exacerbate the negative effect of their diabetes on their mental well-being. If we had been able to control for personality, as well as the unmeasured confounders mentioned at the beginning of this section, the effect sizes and proportions mediated may have been smaller.

We dichotomized several variables for easy interpretation and presentation of results. However, in so doing, there may be a potential loss of granularity, i.e. the level of detail in the data, as the data has been aggregated. Lower granularity is good for providing a broader, more generalized view of the subject, whereas higher granularity offers more specific insights. In this study, the cut-offs decided upon for insomnia and physical activity were “no/subthreshold insomnia” versus “moderate/severe insomnia” and “regular physical activity” versus “less than regular physical activity”. However, severe insomnia and a sedentary level of physical activity could have a more profound effect on depression compared to moderate insomnia and some non-regular physical activity. If the cut-offs had been “no insomnia to moderate insomnia” versus “severe insomnia” and “regular hard physical activity to some physical activity” versus “sedentary”, it may have yielded higher effect sizes and proportions mediated. However, for the purpose of this study, we decided to focus on insomnia and low physical activity as a whole, rather than more extreme cases.

Creating a response category for the missing data in the variable HbA1c, “Not tested”, prevented the exclusion of a large amount of data in the complete case analyses, which could have introduced bias. However, doing so induces risk of bias, as it may distort the effect estimates for observed categories, as the “Not tested” category’s coefficient may absorb variation that would otherwise belong to observed categories.

### Implications for research and practice

The present study suggested that insomnia, and lack of physical activity to a lesser degree, are associated with depression in people with type 2 diabetes. Healthcare workers that work with people with type 2 diabetes may therefore possibly benefit from focusing on insomnia and physical activity in their treatment of, and patient education for, people with type 2 diabetes, in order to promote higher mental well-being. Especially considering that the detection and treatment of sleep disorders are not always part of standard care for people with type 2 diabetes [46]. Higher mental well-being would likely promote better self-management, as studies have suggested that mental state substantially influences diabetes care, with self-managing diabetes being extremely complex for patients who also have severe mental illness [3]. Higher mental well-being and better self-management would also likely promote a longer lifespan, as studies have suggested that unsatisfactory glycemic control is a risk factor for suicidal behavior and suicidal ideation in depressed people with type 2 diabetes [4]. However, further research with better control for confounders and interventions that demonstrate clinical effects is needed.

As stated earlier, the present study did not account for the effect that physical activity might have on insomnia itself, nor the effect of diet, stress, insulin dysfunction, increased inflammation, neuronal alterations, and “male-typical” symptoms of depression on the relationship between diabetes and risk of depression. Further research could examine these associations, carefully distinguishing between the types of stress that increase risk of depression and resilience respectively. Further research could also examine the effect of personality on health behavior.

## Conclusion

People with type 2 diabetes have a higher odds ratio of being at risk for depression compared to people without diabetes. Insomnia and lack of physical activity may mediate the relationship between diabetes and risk of depression, meaning that they may be among the modifiable risk factors that we sought to identify. Healthcare professionals could focus on insomnia and physical activity in their treatment of, and patient education for, people with type 2 diabetes. Future research could focus on the effect of physical activity on insomnia, the confounding effects of diet, stress, insulin dysfunction, inflammation and neuronal alterations, and the effect of personality on the recommended health behavior interventions.

## Data Availability

The HICD cohort is managed by a steering committee at Steno Diabetes Center, Denmark. The committee encourages interested researchers to use this resource. Details about the data application process can be found on the HICD webpage on Steno Diabetes Center Aarhus’ website (https://www.stenoaarhus.dk/research/resources/health-in-central-denmark/). For further inquiries, please contact Kasper Norman (kanoan@rm.dk).

## References

[1] Bo A, et al. Prevalence and correlates of diabetes distress, perceived stress and depressive symptoms among adults with early-onset type 2 diabetes: cross-sectional survey results from the Danish DD2 study. Diabet Med. 2020. 10.1111/dme.14087. 31335989 10.1111/dme.14087

[2] Anderson RJ, et al. The prevalence of comorbid depression in adults with diabetes: a meta-analysis. Diabetes Care. 2001. 10.2337/diacare.24.6.1069. 11375373 10.2337/diacare.24.6.1069

[3] Stenov V, et al. Mental health professionals have never mentioned my diabetes, they don’t get into that: a qualitative study of support needs in adults with type 1 and type 2 diabetes and severe mental illness. Can J Diabetes. 2020. 10.1016/j.jcjd.2020.02.006. 32507647 10.1016/j.jcjd.2020.02.006

[4] Conti C, et al. Clinical characteristics of diabetes mellitus and suicide risk. Front Psychiatry. 2017. 10.3389/fpsyt.2017.00040. 28348533 10.3389/fpsyt.2017.00040 PMC5346593

[5] Fonseca VA. Defining and characterizing the progression of type 2 diabetes. Diabetes Care. 2009. 10.2337/dc09-S301. 19875543 10.2337/dc09-S301 PMC2811457

[6] Wium-Andersen IK, et al. Diabetes, sleep disorders and risk of depression - A Danish register-based cohort study. J Diabetes Complications. 2022. 10.1016/j.jdiacomp.2022.108266. 35932548 10.1016/j.jdiacomp.2022.108266

[7] Aune D, et al. Physical activity and the risk of type 2 diabetes: a systematic review and dose–response meta-analysis. Eur J Epidemiol. 2015. 10.1007/s10654-015-0056-z. 26092138 10.1007/s10654-015-0056-z

[8] Schuch FB, et al. Physical activity and incident depression: A Meta-Analysis of prospective cohort studies. Am J Psychiatry. 2018. 10.1176/appi.ajp.2018.17111194. 29690792 10.1176/appi.ajp.2018.17111194

[9] Bjerg L, et al. Cohort profile: health in central Denmark (HICD) cohort - a register-based questionnaire survey on diabetes and related complications in the central Denmark region. BMJ Open. 2022. 10.1136/bmjopen-2021-060410. 35798528 10.1136/bmjopen-2021-060410 PMC9263900

[10] Rauwerda NL, et al. WHO-5 and BDI-II are acceptable screening instruments for depression in people with diabetes. Diabet Med. 2018. 10.1111/dme.13779. 30019352 10.1111/dme.13779

[11] Topp CW, et al. The WHO-5 Well-Being index: A systematic review of the literature. Psychother Psychosom. 2015. 10.1159/000376585. 25831962 10.1159/000376585

[12] Isaksen AA, et al. Validation of Register-Based diabetes classifiers in Danish data. Clin Epidemiol. 2023. 10.2147/CLEP.S407019. 37180566 10.2147/CLEP.S407019 PMC10167973

[13] Morin CM, et al. The insomnia severity index: psychometric indicators to detect insomnia cases and evaluate treatment response. Sleep. 2011. 10.1093/sleep/34.5.601. 21532953 10.1093/sleep/34.5.601 PMC3079939

[14] Dunbar RIM, et al. Functional benefits of (Modest) alcohol consumption. Adapt Hum Behav Physiol. 2017. 10.1007/s40750-016-0058-4. 10.1007/s40750-016-0058-4 PMC7010365 32104646

[15] Mc Hugh R, McBride O. Self-medicating low mood with alcohol use: examining the role of frequency of alcohol use, quantity consumed and context of drinking. Addict Behav. 2020. 10.1016/j.addbeh.2020.106557. 32721645 10.1016/j.addbeh.2020.106557

[16] Fluharty M, et al. The association of cigarette smoking with depression and anxiety: A systematic review. Nicotine Tob Res. 2017. 10.1093/ntr/ntw140. 27199385 10.1093/ntr/ntw140 PMC5157710

[17] Wickramaratne PJ, et al. Social connectedness as a determinant of mental health: A scoping review. PLoS ONE. 2022. 10.1371/journal.pone.0275004. 36228007 10.1371/journal.pone.0275004 PMC9560615

[18] Hutten E, et al. Loneliness and mental health: the mediating effect of perceived social support. Int J Environ Res Public Health. 2021. 10.3390/ijerph182211963. 34831717 10.3390/ijerph182211963 PMC8619017

[19] Gutiérrez-Rojas L, et al. Prevalence and correlates of major depressive disorder: a systematic review. Braz J Psychiatry. 2020. 10.1590/1516-4446-2020-0650. 32756809 10.1590/1516-4446-2019-0650 PMC7678895

[20] Swetlitz N. Depression’s problem with men. AMA J Ethics. 2021. 10.1001/amajethics.2021.586. 34351273 10.1001/amajethics.2021.586

[21] Lee B, et al. National, State-Level, and County-Level prevalence estimates of adults Aged ≥ 18 years Self-Reporting a lifetime diagnosis of Depression — United States, 2020. MMWR Morb Mortal Wkly Rep. 2023. 10.15585/mmwr.mm7224a1. 37318995 10.15585/mmwr.mm7224a1 PMC10328468

[22] Schaakxs R, et al. Associations between age and the course of major depressive disorder: a 2-year longitudinal cohort study. Lancet Psychiatry. 2018. 10.1016/S2215-0366(18)30166-4. 29887519 10.1016/S2215-0366(18)30166-4

[23] Amiri S. Unemployment associated with major depression disorder and depressive symptoms: a systematic review and meta-analysis. Int J Occup Saf Ergon. 2022. 10.1080/10803548.2021.1954793. 34259616 10.1080/10803548.2021.1954793

[24] Álvaro JL, et al. Unemployment, Self-esteem, and depression: differences between men and women. Span J Psychol. 2019. 10.1017/sjp.2018.68. 30813974 10.1017/sjp.2018.68

[25] Weich S, Lewis G. Poverty, unemployment, and common mental disorders: population based cohort study. BMJ. 1998. 10.1136/bmj.317.7151.115. 9657786 10.1136/bmj.317.7151.115 PMC28602

[26] Bhugra D. Migration and depression. Acta Psychiatr Scand Suppl. 2003. 10.1034/j.1600-0447.108.s418.14.x. 12956818 10.1034/j.1600-0447.108.s418.14.x

[27] Pajediene E, et al. Sex differences in insomnia and circadian rhythm disorders: a systematic review. Med (Kaunas). 2024. 10.3390/medicina60030474. 10.3390/medicina60030474 PMC10971860 38541200

[28] Brewster GS, et al. Insomnia in the older adult. Sleep Med Clin. 2022. 10.1016/j.jsmc.2022.03.004. 35659076 10.1016/j.jsmc.2022.03.004

[29] Maeda M, et al. Association between unemployment and insomnia-related symptoms based on the comprehensive survey of living conditions: a large cross-sectional Japanese population survey. Ind Health. 2019. 10.2486/indhealth.2018-0031. 30918160 10.2486/indhealth.2018-0031 PMC6885596

[30] Chen JH, et al. Marriage, relationship Quality, and sleep among U.S. Older adults. J Health Soc Behav. 2015. 10.1177/0022146515594631. 26272988 10.1177/0022146515594631 PMC4677485

[31] Al-Smadi AM, et al. The prevalence and the predictors of insomnia among refugees. J Health Psychol. 2019. 10.1177/1359105316687631. 28810381 10.1177/1359105316687631

[32] Hu N, et al. Smoking and incidence of insomnia: a systematic review and meta-analysis of cohort studies. Public Health. 2021. 10.1016/j.puhe.2021.07.012. 34507139 10.1016/j.puhe.2021.07.012

[33] Chakravorty S, et al. Alcohol dependence and its relationship with insomnia and other sleep disorders. Alcohol Clin Exp Res. 2016. 10.1111/acer.13217. 27706838 10.1111/acer.13217 PMC7486899

[34] Lawrence C, Marini CM. Loneliness and marital quality as predictors of older adults’ insomnia symptoms. Int J Aging Hum Dev. 2024. 10.1177/00914150231208013. 37849274 10.1177/00914150231208013

[35] Yi M, et al. Unraveling the associations and causalities between glucose metabolism and multiple sleep traits. Front Endocrinol (Lausanne). 2023. 10.3389/fendo.2023.1227372. 38027156 10.3389/fendo.2023.1227372 PMC10660979

[36] Craft BB, et al. Gender differences in exercise habits and quality of life reports: assessing the moderating effects of reasons for exercise. Int J Lib Arts Soc Sci. 2014;2(5):65–76. 27668243 PMC5033515

[37] Alley SJ, et al. Age differences in physical activity intentions and implementation intention preferences. J Behav Med. 2018. 10.1007/s10865-017-9899-y. 29116569 10.1007/s10865-017-9899-y

[38] Van Domelen DR, et al. Employment and physical activity in the U.S. Am J Prev Med. 2011. 10.1016/j.amepre.2011.03.019. 21767720 10.1016/j.amepre.2011.03.019 PMC5221416

[39] Yuan S, et al. The influence of marriage and cohabitation on physical activity among Middle-Aged and older people. J Appl Gerontol. 2024. 10.1177/07334648231203124. 37919978 10.1177/07334648231203124

[40] Wang Q, et al. Physical activity amongst culturally and linguistically diverse communities in australia: a scoping review. Ethn Health. 2023. 10.1080/13557858.2023.2219874. 37271830 10.1080/13557858.2023.2219874

[41] Salin K, et al. Smoking and physical activity trajectories from childhood to midlife. Int J Environ Res Public Health. 2019. 10.3390/ijerph16060974. 30889897 10.3390/ijerph16060974 PMC6466084

[42] Liangpunsakul S, et al. Relationship among alcohol intake, body fat, and physical activity: a population-based study. Ann Epidemiol. 2010. 10.1016/j.annepidem.2010.05.014. 20696406 10.1016/j.annepidem.2010.05.014 PMC2921229

[43] Schrempft S, et al. Associations between social isolation, loneliness, and objective physical activity in older men and women. BMC Public Health. 2019. 10.1186/s12889-019-6424-y. 30651092 10.1186/s12889-019-6424-y PMC6335852

[44] Moser O, et al. Poor glycaemic control is associated with reduced exercise performance and oxygen economy during cardio-pulmonary exercise testing in people with type 1 diabetes. Diabetol Metab Syndr. 2017. 10.1186/s13098-017-0294-1. 29201153 10.1186/s13098-017-0294-1 PMC5697085

[45] Brupbacher G, et al. The effects of exercise on sleep in unipolar depression: a systematic review and network meta-analysis. Sleep Med Rev. 2021. 10.1016/j.smrv.2021.101452. 33667885 10.1016/j.smrv.2021.101452

[46] Schipper SBJ, et al. Sleep disorders in people with type 2 diabetes and associated health outcomes: a review of the literature. Diabetologia. 2021. 10.1007/s00125-021-05541-0. 34401953 10.1007/s00125-021-05541-0 PMC8494668

[47] Pedersen BK, Saltin B. Exercise as medicine - evidence for prescribing exercise as therapy in 26 different chronic diseases. Scand J Med Sci Sports. 2015. 10.1111/sms.12581. 26606383 10.1111/sms.12581

[48] Knapen J, et al. Exercise therapy improves both mental and physical health in patients with major depression. Disabil Rehabil. 2015. 10.3109/09638288.2014.972579. 25342564 10.3109/09638288.2014.972579

[49] Bayes J, et al. The effect of a mediterranean diet on the symptoms of depression in young males (the AMMEND: a mediterranean diet in MEN with depression study): a randomized controlled trial. Am J Clin Nutr. 2022. 10.1093/ajcn/nqac106. 35441666 10.1093/ajcn/nqac106

[50] Scott AJ, et al. Improving sleep quality leads to better mental health: A meta-analysis of randomised controlled trials. Sleep Med Rev. 2021. 10.1016/j.smrv.2021.101556. 34607184 10.1016/j.smrv.2021.101556 PMC8651630

[51] Rotella F, Mannucci E. Diabetes mellitus as a risk factor for depression. A meta-analysis of longitudinal studies. J Clin Psychiatry. 2013. 10.4088/JCP.12r07922. 23419223 10.4088/JCP.12r07922

[52] Vatcheva KP, et al. Multicollinearity in regression analyses conducted in epidemiologic studies. Epidemiol (Sunnyvale). 2016. 10.4172/2161-1165.1000227. 10.4172/2161-1165.1000227 PMC4888898 27274911

[53] Ogurtsova K, et al. IDF diabetes atlas: global estimates of undiagnosed diabetes in adults for 2021. Diabetes Res Clin Pract. 2022. 10.1016/j.diabres.2021.109118. 34883189 10.1016/j.diabres.2021.109118

[54] Koorevaar AML, et al. Big five personality and depression diagnosis, severity and age of onset in older adults. J Affect Disord. 2013. 10.1016/j.jad.2013.05.075. 23820093 10.1016/j.jad.2013.05.075

[55] Shea MT, et al. Does major depression result in lasting personality change? Am J Psychiatry. 1996. 10.1176/ajp.153.11.1404. 8890672 10.1176/ajp.153.11.1404

